# Site-Specific Regulation of Stress Responses Along the Rostrocaudal Axis of the Insular Cortex in Rats

**DOI:** 10.3389/fnins.2022.878927

**Published:** 2022-05-10

**Authors:** Rodrigo A. Tomeo, Lucas Gomes-de-Souza, Ricardo Benini, Lilian L. Reis-Silva, Carlos C. Crestani

**Affiliations:** Laboratory of Pharmacology, School of Pharmaceutical Sciences, São Paulo State University (UNESP), Araraquara, Brazil

**Keywords:** cardiovascular function, Fos, topography, prefrontal cortex, restraint stress, sympathetic activity

## Abstract

The insular cortex (IC) has been described as a part of the central network implicated in the integration and processing of limbic information, being related to the behavioral and physiological responses to stressful events. Besides, a site-specific control of physiological functions has been reported along the rostrocaudal axis of the IC. However, a functional topography of the IC in the regulation of stress responses has never been reported. Therefore, this study aimed to investigate the impact of acute restraint stress in neuronal activation at different sites along the rostrocaudal axis of the IC. Furthermore, we evaluated the involvement of IC rostrocaudal subregions in the cardiovascular responses to acute restraint stress. We observed that an acute session of restraint stress increased the number of Fos-immunoreactive cells in the rostral posterior region of the IC, while fewer activated cells were identified in the anterior and caudal posterior regions. Bilateral injection of the non-selective synaptic inhibitor CoCl_2_ into the anterior region of the IC did not affect the blood pressure and heart rate increases and the sympathetically mediated cutaneous vasoconstriction to acute restraint stress. However, synaptic ablation of the rostral posterior IC decreased the restraint-evoked arterial pressure increase, whereas tachycardia was reduced in animals in which the caudal posterior IC was inhibited. Taken together, these pieces of evidence indicate a site-specific regulation of cardiovascular stress response along the rostrocaudal axis of the IC.

## Introduction

The responses evoked by stressful stimuli are mediated by limbic networks in the central nervous system involving cortical, subcortical, hypothalamic, and brainstem structures (Dampney, 2015; Myers, 2017; Schaeuble and Myers, 2022). In this sense, the insular cortex (IC) has been described as a part of the brain network implicated in the integration and processing of limbic information in humans subjects and animals (Augustine, 1996; Nieuwenhuys, 2012; Uddin, 2014; Gogolla, 2017). The IC has been proposed to integrate limbic information from several cortical and subcortical areas and, in turn, to control physiological and behavioral responses during aversive situations through connections with hypothalamic and brainstem structures (Augustine, 1996; Nieuwenhuys, 2012; Uddin, 2014; Gogolla, 2017). Previous studies showed an involvement of the IC in the cardiovascular responses to aversive threats. For instance, it was identified that non-selective synaptic inhibition of the IC of rats caused by local microinjection of CoCl_2_ reduced the cardiovascular responses caused by both conditioned (i.e., contextual conditioned response) and unconditioned (i.e., restraint stress) aversive stimuli (Alves et al., 2010, 2013).

A site-specific control of the cardiovascular function under basal conditions (i.e., non-stressed) along the rostrocaudal axis of the IC has been described in rodents (Verberne and Owens, 1998; Marins et al., 2016, 2021). Indeed, stimulation of the IC (electrical or chemical with excitatory amino acids) evidenced that activation of anterior regions evoked mainly decreases in arterial pressure (Hardy and Holmes, 1988; Hardy and Mack, 1990; Sun, 1992). Conversely, stimulation of posterior IC caused either a pressor response accompanied by tachycardia or a reduction of both blood pressure and heart rate (HR) (Verberne and Owens, 1998). It was identified that the sites in the posterior IC causing pressor and tachycardiac responses were located more rostrally than those evoking arterial pressure and HR decreases (Verberne and Owens, 1998). Recent results have confirmed a site-specific control of cardiovascular function under basal conditions in the posterior region of the IC (Marins et al., 2016, 2021).

Despite these pieces of evidence, a rostrocaudal organization in the control of stress responses by the IC has never been investigated. Thus, even though previous studies in humans and animals have identified that several stressful stimuli (including restraint stress in rats) caused neuronal activation in the IC (Cullinan et al., 1995; Yokoyama and Sasaki, 1999; Gianaros et al., 2007; Cechetto, 2014), we did not identify studies that have systematically evaluated stress-induced changes on the number of activated cells along the rostrocaudal axis of the IC. Furthermore, despite evidence of a rostrocaudal organization in cardiovascular control by the IC at rest, evaluation of a functional topography of the IC in the regulation of cardiovascular and autonomic changes to stressful stimuli was never performed. Therefore, in this study we investigated the effect of acute restraint stress on neuronal activation of anterior, rostral posterior, and caudal posterior subregions of the IC. Besides, we evaluated the role of these specific sites along the rostrocaudal axis of the IC in the cardiovascular changes caused by acute restraint stress.

## Materials and Methods

### Animals

This study used 76 male Wistar rats (230–250 g; 60 days old) provided by the Central Animal Facility of the UNESP (Botucatu, SP, Brazil). All rats had free access to standard laboratory food and water and were kept in acclimatized rooms (temperature at 24 ± 2°C and humidity at 50 ± 5%) with lights on between 7:00 and 19:00 h. Experimental procedures were approved by the Institutional Ethical Committee for Use of Animals (approval # 38/2018).

### Experimental Design

Animals were brought to the trial room in their own home-cages and were allowed to adapt to the experimental room conditions (at least 60 min) before beginning the tests. The trial room was temperature-controlled (24°C) and isolated from the other rooms.

#### Effect of Acute Restraint Stress on the Number of Fos-Positive Cells Along the Rostrocaudal Axis of the Insular Cortex

Different sets of rats were subjected to either a 60-min session of restraint stress (restraint group) or kept at rest in the home-cage (control group). Thirty minutes following the termination of the stress session (i.e., 90 min after the beginning of the restraint session), the animals of control and restraint groups were anesthetized [urethane, 1.2 g/kg, intraperitoneally (i.p.)], perfused, and the brain was removed for assessment of the number of Fos-positive cells in the anterior, rostral posterior, and caudal posterior subregions of the IC. The definitions of the subregions were based on previous studies (Saper, 1982; Oppenheimer and Cechetto, 1990, Oppenheimer and Cechetto, 2016; Marins et al., 2016, 2020).

#### Effect of Non-selective Synaptic Inhibition Along the Rostrocaudal Axis of the Insular Cortex on Cardiovascular and Tail Skin Temperature Responses to Acute Restraint Stress

Each animal was connected to the cardiovascular recording system in its respective home-cage. Then, baseline arterial pressure and HR recording was performed for a period of at least 20 min. Later, different sets of animals received bilateral microinjection of the non-selective synaptic inhibitor CoCl_2_ (1 mM/100 nl) or vehicle (saline, 100 nl) (Alves et al., 2010) into either the anterior, rostral posterior, or caudal posterior subregions of the IC. Ten min after IC treatment, rats underwent a 60-min session of restraint stress (restraint period) (Alves et al., 2010, 2014). Following restraint termination, the animals returned to the respective home-cage and the cardiovascular parameters were recorded for a 60-min period (recovery period).

Cardiovascular recordings (i.e., arterial pressure and HR) started at least 20 min before the restraint stress session and were performed throughout the restraint and recovery periods. Sympathetically mediated vasoconstriction of cutaneous beds during aversive threats reduces skin temperature (Blessing, 2003; Vianna and Carrive, 2005). Therefore, the drop in tail skin temperature during restraint was measured as an indirect measure of the sympathetic response to cutaneous beds (Oliveira et al., 2015; Gomes-de-Souza et al., 2021). Measurements of tail skin temperature were performed at 5 and 0 min before restraint for basal values; 5, 10, 20, 40, and 60 min during restraint, as well as 10, 20, 30, 40, 50, and 60 min during the recovery phase (Benini et al., 2019; Barretto-de-souza et al., 2021).

### Acute Restraint Stress

For restraint stress, animals were kept for 60 min into a cylindrical plastic tube, as previously described (Alves et al., 2010, 2014). Animals were subjected to only one restraint session to avoid the habituation process (Benini et al., 2019; Santos et al., 2020).

### Immunohistochemistry

The procedures for the assessment of Fos-positive cells were as previously described (Gomes-de-Souza et al., 2021). Briefly, 90 min after the onset of the restraint stress (i.e., 30 min following the termination of the restraint stress session), the rats were anesthetized (urethane, 1.2 g/kg, i.p.) and subjected to perfusion. Later, the brain was obtained and fixed [paraformaldehyde and 30% sucrose solution in phosphate-buffered saline (PBS)]. Then, the brains were sectioned into 35-μm frontal cuts (CM1900, Leica, Wetzlar, Germany) and the slices containing the anterior, rostral posterior, and caudal posterior subregions of the IC were obtained. The IC subregions were determined according to Paxinos and Watson (1997). The slices were washed (PBS) and incubated for 1 h at room temperature in a blocking solution (0.25% Triton X-100 and 3% goat serum in PBS). Next, the slices were incubated for 24 h at 4°C with an anti-Fos primary antibody produced in rabbits (cat.# 5348; dilution 1:2,000; Cell Signaling, Danvers, MA, United States). Later, the slices were washed (PBS) and incubated for 2 h at room temperature with biotinylated antirabbit secondary antibody (cat.# BA-1000; dilution 1:600; Vector Laboratories, Burlingame, CA, United States). The slices were then washed (PBS) and incubated for 1 h in avidin–biotin–peroxidase solution [cat.# PK-6100; Vectastain Elite ABC-HRP Kit, Peroxidase (Standard); Vector Laboratories, Burlingame, CA, United States]. Then, the slices were washed and incubated for 7 min in 3, 3’-diaminobenzidine tetrahydrochloride hydrate (cat.# D5637, Sigma-Aldrich, St Louis, MO, United States). Finally, the slices were washed and mounted on gelatinized slides.

Images of the right and left hemispheres were captured in each slice, and the analysis was performed in at least two slices per animal. The number of Fos-immunoreactive cells was calculated from a fixed area of each IC subregion using the ImageJ software. The results are presented as the number of Fos-immunoreactive neurons/mm^2^.

### Stereotaxic Surgery

Five days before the experiments, rats were anesthetized with tribomoethanol at a dose of 250 mg/kg i.p. The animal’s head was shaved and then fixed to a stereotaxic apparatus (Stoelting, Wood Dale, IL, United States), and the surgical field was cleaned with 70% alcohol and iodine. A solution of lidocaine with vasoconstrictor (2% lidocaine + 3% phenylephrine, Harvey Química Farmacêutica, Catanduva, SP, Brazil) was administrated for scalp anesthesia and bleeding reduction. Then, the skull was exposed, and the region was cleaned with saline and hydrogen peroxide (10%). Bilateral cannulas were then directed to either the anterior, rostral posterior, or caudal posterior subregions of the IC. The coordinates used for implanting the guide cannulas were anteroposterior, +11.7 mm (anterior IC); +9.1 mm (rostral posterior IC) or +7.0 mm (caudal posterior IC) in relation to the interaural; lateral, +4 mm (anterior IC), +5 mm (rostral posterior IC), or +6.2 mm (caudal posterior IC) from the middle suture; vertical, −4.5 mm (anterior IC), −5.0 mm (rostral posterior IC), or −6.0 mm (caudal posterior IC) in relation to the skull (Paxinos and Watson, 1997). Cannulas were implanted without lateral angulation, and incisor bar was set at −3.2 mm. Two holes were made in the skull using a dental drill through which the bilateral cannulas (26 G, 12 mm) were introduced. The cannulas were fixed to the skull with a self-curing acrylic resin (Simples, DFL, Ind. Com., Rio de Janeiro, RJ, Brazil) and stainless-steel screws were previously implanted in the skull to fix the resin. A 0.2-mm-diameter mandrel was introduced into the cannula to prevent it from clogging. At the end of the surgery, the rats were treated with a veterinary antibiotic intramuscularly (560 mg/ml/kg) and the non-steroidal anti-inflammatory flunixin meglumine by the subcutaneous route (0.5 mg/ml/kg).

### Cannulation of Femoral Artery

Twenty-four hours before the trial, the rats were again anesthetized (tribromoethanol, 250 mg/kg, i.p.) and a polyethylene catheter was implanted into the femoral artery for arterial pressure and HR recording. The cannula consisted of a PE-10 polyethylene segment (4–5 cm) welded to a PE-50 polyethylene segment (12–13 cm) (Clay Adams, Parsippany, NJ, United States). The cannulas were filled with a saline solution containing anticoagulant (50 IU/ml of heparin; Hepamax-S, Blausiegel, Cotia, SP, Brazil) and occluded with a metal pin before the implantation. The catheter was exteriorized in the animal’s dorsal region and fixed to the skin by surgical suture.

Animals were individually housed during the postoperative period and trials. At the end of the surgery, the animals were treated with the non-steroidal anti-inflammatory flunixin meglumine by the subcutaneous route (0.5 mg/ml/kg).

### Arterial Pressure and Heart Rate Recording

Cardiovascular parameters (i.e., arterial pressure and HR) were recorded as previously described (Almeida et al., 2015; Oliveira et al., 2015). Briefly, the arterial catheter (implanted into the femoral artery) was connected to a pressure transducer and pulsatile arterial pressure (PAP) was recorded using an amplifier connected to an acquisition system (ADInstruments, Australia). The mean arterial pressure (MAP) and HR were calculated from the PAP signal.

### Tail Skin Temperature Recording

Tail skin temperature recording was performed as previously described (Oliveira et al., 2015; Gomes-de-Souza et al., 2021). Briefly, the recordings were performed using a thermal camera (IRI4010, InfraRed Integrated Systems Ltd., Northampton, United Kingdom) and the temperature was obtained at five sites along the tail, and then the mean was obtained for each record (Oliveira et al., 2015; Gomes-de-Souza et al., 2021).

### Drugs and Solutions

CoCl_2_ (non-selective synaptic inhibitor) (cat. # 232696; Sigma-Aldrich, St Louis, Missouri, United States), tribromoethanol (cat. # T48402, Sigma-Aldrich), and urethane (cat. # U2500, Sigma-Aldrich) were dissolved in saline solution (0.9% NaCl). The antibiotic (Pentabiotico, Fort-Dodge, Campinas, SP, Brazil) and the non-steroidal anti-inflammatory flunixin meglumine (Banamine, Schering Plough, Cotia, SP, Brazil) were used as supplied by the manufacturers.

### Intra-Brain Microinjection

The injection needles (33 G, Small Parts, Miami Lakes, FL, United States) used for intra-brain pharmacological treatment (1 mm longer than the guide-cannulas) were connected to a microsyringe (7002-KH, Hamilton Co., Reno, NV, United States) using a polyethylene tube (PE-10). Vehicle and CoCl_2_ were microinjected in a final volume of 100 nl/side (Alves et al., 2010; Marins et al., 2016).

### Determination of Intra-Brain Microinjection Sites

Following the termination of each experiment, the rats subjected to intra-brain pharmacological treatment were anesthetized (urethane, 1.2 g/kg, i.p.) and 1% Evan’s blue dye was bilaterally microinjected (100 nl/side) in the brain to determine the injection sites. Then, the brain was removed, fixed in 10% formalin, and subsequently sectioned into 50-μm frontal cuts (CM1900, Leica, Wetzlar, Germany). Microinjection sites were determined in a microscope (Zeiss Axioskop 2) based on Paxinos and Watson (1997).

### Statistical Analysis

Data were expressed as mean ± standard error of the mean (SEM). Baseline parameters of MAP, HR, and tail skin temperature, as well as the number of Fos-immunoreactive cells, were analyzed using Student’s *t*-test. Two-way analysis of variance (ANOVA), with treatment as an independent factor and time as a repeated measure, was used to analyze the time-course curves. The mean of cardiovascular and tail skin temperature responses during the restraint and recovery periods was also obtained and analyzed using the two-way ANOVA (treatment as an independent factor and time as a repeated measure). *P* < 0.05 was assumed to be significant.

## Results

**FIGURE 1:**
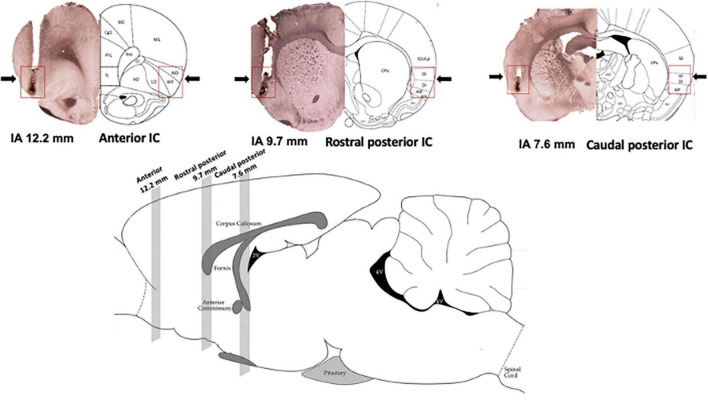
Location of the subregions along the rostrocaudal axis of the insular cortex (IC). (Top) Photomicrograph of coronal brain sections depicting the microinjection sites of representative animals (left hemisphere) and diagrammatic representations modified from the rat brain atlas of Paxinos and Watson (1997) (right hemisphere) indicating the anterior [interaural (IA) 12.7 mm], rostral posterior (IA 9.7 mm), and caudal posterior (IA 7.6 mm) subregions of the IC. Arrows and red squares indicate the microinjection site and IC region. (Bottom) Representation of a sagittal section of the rat brain modified from the rat brain atlas of Paxinos and Watson (1997) indicating the position of the anterior (12.7 mm), rostral posterior (9.7 mm), and caudal posterior (7.6 mm) subregions of the IC along the rat brain. The numbers indicate the IA distance.

Diagrammatic representations of coronal sections (Paxinos and Watson, 1997) as well as photomicrographs of coronal brain sections showing microinjection sites of representative animals in the anterior, rostral posterior, and caudal posterior subregions of the IC are presented in Figure 1. Figure 1 also presents the representation of a sagittal section of the rat brain (Paxinos and Watson, 1997), indicating the rostrocaudal position of the IC subregions along the rat brain.

### Effect of Restraint Stress on the Number of Fos-Positive Cells Along the Rostrocaudal Axis of the Insular Cortex

**FIGURE 2:**
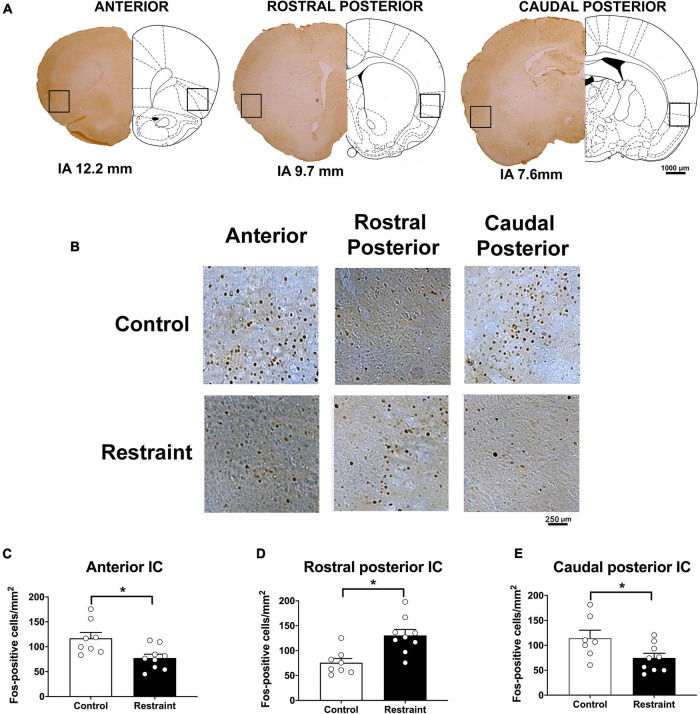
Effect of restraint stress on the number of Fos-positive cells along the rostrocaudal axis of the insular cortex (IC) **(A)**. Representative coronal sections indicating the IC locations where the Fos-positive cells were quantified **(B)**. Representative coronal sections showing Fos-positive cells of representative animals in the anterior (left), rostral posterior (middle), and caudal posterior (right) subregions of the IC in control animals (top) and rats subjected to an acute 60-min session of restraint stress (bottom). **(C–E)** Number of Fos-positive cells in the anterior **(C)**, rostral posterior **(D)**, and caudal posterior **(E)** regions of the IC in control animals (white bars, *n* = 8) and rats subjected to an acute 60-min session of restraint stress (black bars, *n* = 9). The bars represent the mean ± SEM. *p < 0.05, Student’s *t*-test.

Figures 2A,B also presents coronal brain sections of representative animals showing Fos-immunoreactive cells in the anterior, rostral posterior, and caudal posterior subregions of the IC of control animals and rats subjected to restraint stress, as well as an indication of the IC locations where the analysis was performed. Analysis indicated that exposure to a 60-min session of restraint stress (*n* = 9) decreased the number of Fos-positive cells in the anterior (*t* = 2.44, *df* = 15, *p* = 0.0276) (Figure 2C) and caudal posterior (*t* = 2.27, *df* = 15, *p* = 0.0388) (Figure 2E) subregions of the IC (Figure 2) when compared with control animals (*n* = 8). Conversely, increased number of Fos-immunoreactive cells was found in the rostral posterior IC following acute restraint stress (*t* = 2.95, *df* = 15, *p* = 0.0106) (Figure 2D).

### Effect of Non-selective Synaptic Inhibition Along the Rostrocaudal Axis of the Insular Cortex on Basal Parameters of Mean Arterial Pressure, Heart Rate, and Tail Skin Temperature

Analysis of the basal parameters indicated that bilateral microinjection of the non-selective synaptic inhibitor CoCl_2_ (1 mM/100 nl) into either the rostral posterior or caudal posterior subregions of the IC did not affect the baseline values of MAP, HR, and tail skin temperature (Table 1). However, analysis of basal parameters in animals that received CoCl_2_ into the anterior IC indicated enhanced tail skin temperature values when compared with vehicle-treated animals, without changes of MAP and HR (Table 1).

**TABLE 1:** Basal parameters (i.e., pre-stress values) of mean arterial pressure (MAP), heart rate (HR), and tail skin temperature (T) after pharmacological treatment of either the anterior, rostral posterior, or caudal posterior regions of the insular cortex (IC) with the non-selective synaptic inhibitor CoCl_2_ or vehicle.

Groups	n	MAP (mmHg)	HR (bpm)	T (°C)
**Anterior IC**				
Vehicle	8	98 ± 1	370 ± 12	29.3 ± 0.4
CoCl_2_	9	100 ± 2	344 ± 7	31.5 ± 0.6*
		*t* = 1.2, df = 15	*t* = 1.9, df = 15	*t* = 2.9, df = 15
		*P* = 0.2373	*P* = 0.0668	*P* = 0.0096
**Rostral posterior IC**				
Vehicle	11	102 ± 2	351 ± 5	29.6 ± 0.6
CoCl_2_	12	106 ± 2	355 ± 12	30.2 ± 0.5
		*t* = 1.2, df = 21	*t* = 0.3, df = 21	*t* = 0.8, df = 21
		*P* = 0.2439	*P* = 0.7458	*P* = 0.4481
**Caudal posterior IC**				
Vehicle	10	101 ± 2	354 ± 5	28.9 ± 0.1
CoCl_2_	9	102 ± 3	378 ± 12	30.2 ± 0.4
		*t* = 0.4, df = 17	*t* = 2.0, df = 17	*t* = 0.9, df = 17
		*P* = 0.6942	*P* = 0.0542	*P* = 0.4559

*Values are mean ± SEM, Student’s t-test.*

*p < 0.05.

### Effect of Non-selective Synaptic Inhibition Along the Rostrocaudal Axis of the Insular Cortex on Cardiovascular and Tail Skin Temperature Changes Evoked by Acute Restraint Stress Anterior Insular Cortex

**FIGURE 3:**
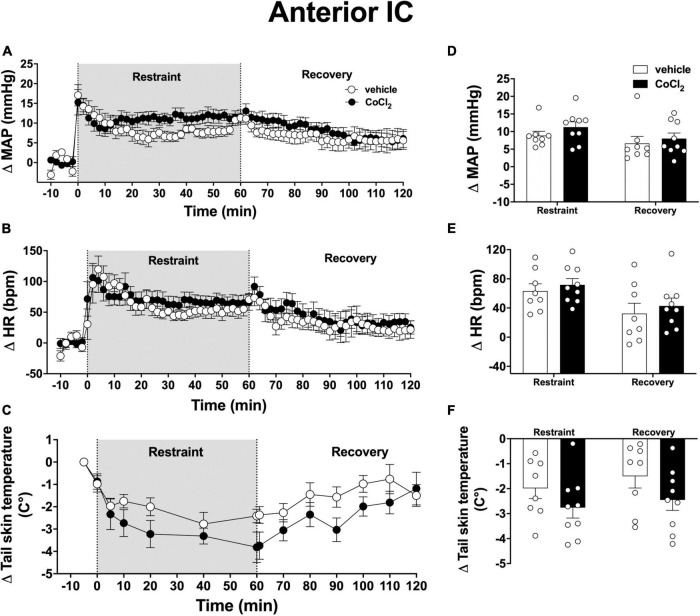
Effect of bilateral microinjection of the non-selective synaptic inhibitor CoCl_2_ into the anterior subregion of the insular cortex (IC) on mean arterial pressure (MAP), heart rate (HR), and tail skin temperature responses evoked by an acute 60-min session of restraint stress. **(A–C)** Time-course curves of changes on MAP (Δ MAP), HR (Δ HR), and tail skin temperature (Δ tail skin temperature) evoked by acute restraint stress in animals treated bilaterally into the anterior subregion of the IC with vehicle (saline, 100 nl, *n* = 8) or the non-selective synaptic inhibitor CoCl_2_ (1 mM/100 nl, *n* = 9). Shaded area indicates the restraint stress period. Circles represent the mean ± SEM. Two-way ANOVA. **(D–F)** Mean of Δ MAP, Δ HR, and Δ tail skin temperature during the restraint and recovery periods in animals treated bilaterally into the anterior IC with vehicle (*n* = 8) or CoCl_2_ (*n* = 9). Bars represent the mean ± SEM. Two-way ANOVA.

Analysis of the time-course curves indicated that an acute 60-min session of restraint stress increased MAP [time factor: *F*_(65, 975)_ = 14.52; *p* < 0.0001] and HR [time factor: *F*_(65, 975)_ = 11.06; *p* < 0.0001], and decreased tail skin temperature [time factor: *F*_(13, 195)_ = 16.79; *p* < 0.001] (Figures 3A–C). Treatment of the anterior subregion of the IC with the non-selective synaptic inhibitor CoCl_2_ (1 mM/100 nl, *n* = 9) did not affect the restraint-evoked increase in MAP [*F*_(1, 15)_ = 0.90, *p* = 0.3633] and HR [*F*_(1, 15)_ = 0.3, *p* = 0.5691], as well as the drop in tail skin temperature [*F*_(1, 15)_ = 2.23, *p* = 0.1608] when compared with animals treated locally with vehicle (100 nl, *n* = 8) (Figures 3A–C). We identified an interaction between time and treatment for MAP [*F*_(65, 975)_ = 1.82, *p* = 0.0004], but not for HR [*F*_(65, 975)_ = 0.68, *p* = 0.9786] and tail skin temperature [*F*_(13, 195)_ = 1.75, *p* = 0.0624] values.

Analysis of mean responses during the restraint and recovery periods indicated differences in the values during restraint and recovery periods for MAP [time factor: *F*_(1, 15)_ = 13.21, *p* = 0.0025), HR [time factor: *F*_(1, 15)_ = 25.40, *p* = 0.0002], and tail skin temperature [time factor: *F*_(1, 15)_ = 5.33, *p* = 0.0363] (Figures 3D–F). However, two-way ANOVA did not indicate neither effect of the bilateral microinjection of CoCl_2_ into the anterior IC [MAP: *F*_(1, 15)_ = 0.80, *p* = 0.3879; HR: *F*_(1, 15)_ = 0.69, *p* = 0.4865; and skin temperature: *F*_(1, 15)_ = 2.38, *p* = 0.1213]nor treatment × time interaction [MAP: *F*_(1, 15)_ = 0.47, *p* = 0.5041; HR: *F*_(1, 15)_ = 0.01, *p* = 0.9206; and skin temperature: *F*_(1, 15)_ = 0.59, *p* = 0.6354] for all measurements (Figures 3D–F).

### Rostral Posterior Insular Cortex

**FIGURE 4:**
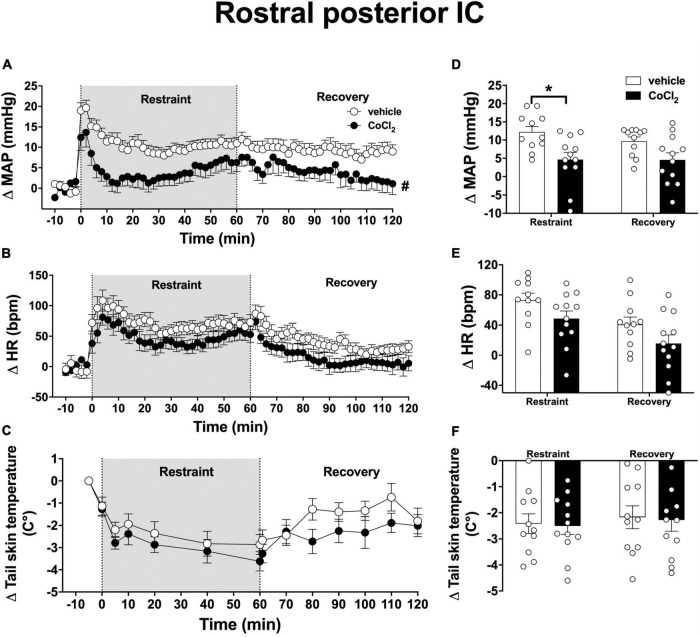
Effect of bilateral microinjection of the non-selective synaptic inhibitor CoCl_2_ into the rostral posterior subregion of the insular cortex (IC) on mean arterial pressure (MAP), heart rate (HR), and tail skin temperature responses evoked by an acute 60-min session of restraint stress. **(A–C)** Time-course curves of changes on MAP (Δ MAP), HR (Δ HR), and tail skin temperature (Δ tail skin temperature) evoked by acute restraint stress in animals treated bilaterally into the rostral posterior IC with vehicle (saline, 100 nl, *n* = 11) or the non-selective synaptic inhibitor CoCl_2_ (1 mM/100 nl, *n* = 12). Circles represent the mean ± SEM. ^#^*p* < 0.05 over the entire restraint and recovery period compared to vehicle group. Two-way ANOVA. **(D–F)** Mean of Δ MAP, Δ HR, and Δ tail skin temperature during the entire restraint and recovery periods in animals treated bilaterally into the rostral posterior IC with vehicle (*n* = 11) or CoCl_2_ (*n* = 12). Bars represent the mean ± SEM. *p < 0.05 compared to vehicle group. Two-way ANOVA followed by Bonferroni *post-hoc* test.

Analysis of the time-course curves indicated that acute restraint stress increased MAP [time factor: *F*_(65, 1, 365)_ = 9.5, *p* < 0.0001] and HR [time factor: *F*_(65, 1, 365)_ = 12.3, *p* < 0.0001] and decreased the tail skin temperature values [time factor: *F*_(13, 273)_ = 15.8, *p* < 0.0001] (Figures 4A–C). Treatment of the rostral posterior subregion of the IC with CoCl_2_ (1 mM/100 nl, *n* = 12) decreased the restraint-evoked MAP increase [*F*_(1, 21)_ = 5.3, *p* = 0.0313], without affecting the tachycardia [*F*_(1, 21)_ = 4.1, *p* = 0.0561] and the reduction in tail skin temperature [*F*_(1, 21)_ = 0.05, *p* = 0.8411]; when compared with animals treated locally with vehicle (100 nl, *n* = 11) (Figures 4A–C). Analysis also indicated an interaction between time and treatment for MAP [*F*_(65, 1, 365)_ = 1.63, *p* = 0.0013], but not for HR [*F*_(65, 1, 365)_ = 0.6, *p* = 0.9827] and tail skin temperature [*F*_(13, 273)_ = 0.4, *p* = 0.9708].

Analysis of the mean Δ MAP during the restraint and recovery periods indicated effect of the CoCl_2_ microinjection into the rostral posterior IC [*F*_(1, 21)_ = 8.47, *p* = 0.0081], but without significant differences in values during restraint and recovery periods [time factor: *F*_(1, 21)_ = 2.62, *p* = 0.1216] and interaction between treatment and time [*F*_(1, 21)_ = 2.35, *p* = 0.1414] (Figure 4D). Bonferroni *post-hoc* analysis revealed that MAP values during the restraint (*p* = 0.0045), but not during the recovery period (*p* = 0.0603), were reduced in animals treated with CoCl_2_ in the rostral posterior IC (Figure 4D). Analysis of HR values indicated effect of time [*F*_(1, 21)_ = 18.12, *p* = 0.0004], but without influence of CoCl_2_ treatment [*F*_(1, 21)_ = 4.20, *p* = 0.0531] and interaction between the factors [*F*_(1, 21)_ = 0.005, *p* = 0.9481] (Figure 4E). Analysis of tail skin temperature did not indicate effect of either time [*F*_(1, 21)_ = 1.29, *p* = 0.2707] or treatment [*F*_(1, 21)_ = 0.03, *p* = 0.8557], or interaction between the factors [*F*_(1, 21)_ = 0.002, *p* = 0.9653] (Figure 4F).

### Caudal Posterior Insular Cortex

**FIGURE 5:**
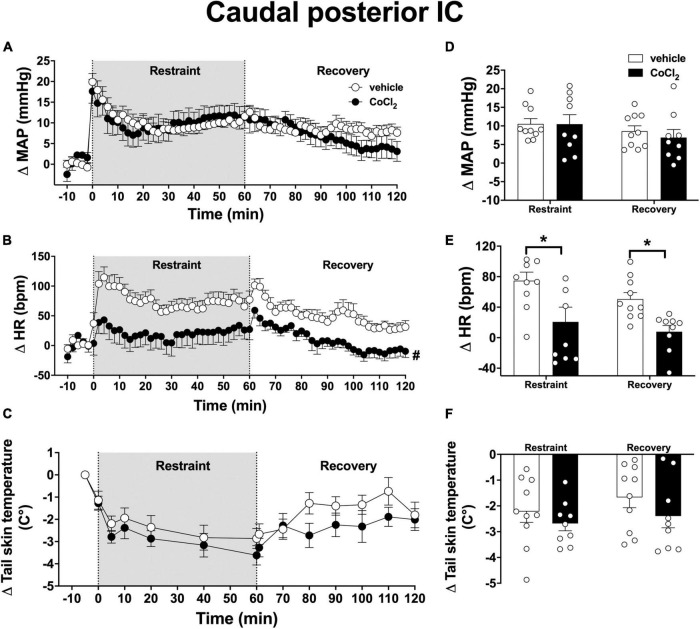
Effect of bilateral microinjection of the non-selective synaptic inhibitor CoCl_2_ into the caudal posterior subregion of the insular cortex (IC) on mean arterial pressure (MAP), heart rate (HR), and tail skin temperature responses evoked by an acute session of restraint stress. **(A–C)** Time-course curves of changes on MAP (Δ MAP), HR (Δ HR), and tail skin temperature (Δ tail skin temperature) evoked by acute restraint stress in animals treated bilaterally into the caudal posterior IC with vehicle (saline, 100 nl, *n* = 10) or the non-selective synaptic inhibitor CoCl_2_ (1 mM/100 nl, *n* = 9). Shaded area indicates the restraint stress period. Circles represent the mean ± SEM. ^#^*p* < 0.05 over the entire restraint and recovery period compared to vehicle group. Two-way ANOVA. **(D–F)** Mean of Δ MAP, Δ HR, and Δ tail skin temperature during the entire restraint and recovery periods in animals treated bilaterally into the caudal posterior IC with vehicle (*n* = 10) or CoCl_2_ (1 nmol/100 nl, *n* = 9). Bars represent the mean ± SEM. *p < 0.05 compared to vehicle group. Two-way ANOVA followed by Bonferroni *post-hoc* test.

Analysis of the time-course curves indicated that acute restraint stress caused an increase on MAP [time factor: *F*_(65, 1,105)_ = 11.6, *p* < 0.0001] and HR [time factor: *F*_(65, 1,105)_ = 6.3, *p* < 0.0001] and decreased the values of tail skin temperature [time factor: *F*_(13, 221)_ = 13.5, *p* < 0.0001] (Figures 5A–C). Bilateral microinjection of CoCl_2_ (1 mM/100 nl, *n* = 9) into the caudal posterior IC decreased the tachycardia to restraint stress [*F*_(1, 17)_ = 11.3, *p* = 0.0036], without affecting the MAP [*F*_(1, 17)_ = 0.11, *p* = 0.7462] and tail skin temperature [*F*_(1, 17)_ = 1.4, *p* = 0.2640] responses when compared to vehicle-treated animals (100 nl, *n* = 10) (Figures 5A–C). Analysis did not indicate a time × treatment interaction for either MAP [*F*_(65, 1,105)_ = 1.4, *p* = 0.0686], HR [*F*_(65, 1,105)_ = 1.1, *p* = 0.2151], or tail skin temperature [*F*_(13, 221)_ = 0.9, *p* = 0.5022].

Analysis of the mean Δ HR during the restraint and recovery periods indicated effect of the microinjection of CoCl_2_ into the caudal posterior IC [*F*_(1, 17)_ = 11.85, *p* = 0.0029], without differences in values during restraint and recovery periods [time factor: *F*_(1, 17)_ = 3.85, *p* = 0.0602] and interaction between treatment and time [*F*_(1, 17)_ = 0.23, *p* = 0.4569] (Figure 5E). Bonferroni *post-hoc* analysis revealed that HR values during restraint (*p* = 0.0076) and recovery (*p* = 0.0294) periods were reduced in animals treated with CoCl_2_ into the caudal posterior IC (Figure 5E). Analysis of MAP values indicated effect of time [*F*_(1, 17)_ = 8.63, *p* = 0.0092], without treatment influence [*F*_(1, 17)_ = 0.12, *p* = 0.7243] and interaction between the factors [*F*_(1, 17)_ = 0.75, *p* = 0.3964] (Figure 5D). Analysis of tail skin temperature did not indicate effect of either time [*F*_(1, 17)_ = 3.57, *p* = 0.0763] or treatment [*F*_(1, 17)_ = 1.32, *p* = 0.2683], or interaction between the factors [*F*_(1, 17)_ = 0.35, *p* = 0.5648] (Figure 5F).

## Discussion

This study reports the effect of restraint stress in neuronal activation along the rostrocaudal axis of the IC. We also document the role of the anterior, rostral posterior, and caudal posterior subregions of the IC in cardiovascular and tail skin temperature changes to acute restraint stress. We identified that acute restraint stress exposure decreased the number of Fos-positive cells in the anterior and caudal posterior regions of the IC, whereas enhanced neuronal activation was observed in the rostral posterior subregion. Analysis of the cardiovascular responses indicated a role of the rostral posterior IC in the pressor response to restraint stress, whereas the caudal posterior region seems to be involved in tachycardia. Non-selective synaptic inhibition in the anterior IC did not affect any parameter analyzed. Table 2 summarizes the main findings reported in this study.

**TABLE 2:** Summary of the main findings obtained in this study.

	Insular Cortex (IC)		
	
	Anterior	Rostral posterior	Caudal posterior
**Effect of restraint stress**			
Fos-positive cells	**↓**	**↑**	**↓**
**Effect of local CoCl_2_ microinjection**			
Pressor effect	=	**↓**	=
Tachycardia	=	=	**↓**
Cutaneous vasoconstriction	=	=	=

***↓**, decrease; **↑**, increase; =, no effect.*

Alves et al. (2010) first documented an involvement of the IC in the cardiovascular changes to restraint stress. They observed that bilateral injection of the non-selective synaptic inhibitor CoCl_2_ into sites located in the anterior region of the IC decreased both the MAP and HR increases observed during restraint stress. Conversely, the results obtained by us did not indicate a role of the anterior subregion of the IC in restraint-evoked cardiovascular changes. However, the microinjection sites in the study by Alves et al. (2010) reached slightly more caudal regions of the anterior IC (11.7–12.7 mm from interaural) when compared to those in this study (12.2–12.7 mm from interaural). Thus, an explanation for the discrepancy might be the existence of a site-specific control along the rostrocaudal axis of the anterior portion of the IC, so that control of cardiovascular changes to stress occurs in caudal regions of the anterior IC. However, a possible characterization in this sense using intra-brain microinjection approach is difficult since the extent of the anterior region of the IC is short, which would entail very close microinjections for a rostrocaudal analysis. Thus, although our findings do not indicate an involvement of the anterior IC in the cardiovascular responses to restraint stress, we cannot completely exclude its participation. Besides, the evidence documented here that restraint stress decreased the neuronal activity in anterior IC indicates that the sites of the anterior region explored in this study might be involved in responses to aversive threats.

Our data evidenced a site-specific control of MAP and HR responses during restraint stress in the posterior portion of the IC. Indeed, while inhibition of the rostral posterior region decreased specifically the pressor response to restraint stress, the caudal posterior IC was implicated in the tachycardia. Analysis of Fos-immunoreactive neurons indicated that involvement of rostral posterior region in the restraint-evoked pressor response is mediated by neuronal activation of this region, as evidenced by the enhanced number of Fos-positive cells in animals subjected to restraint stress. Previous studies indicated that the arterial pressure increase caused by restraint stress is mainly due to vasomotor sympathoexcitation (Dos Reis et al., 2014). In this sense, our findings are supported by previous evidence from studies in animals at rest (i.e., non-stressed) in which vasomotor sympathoexcitatory responses were evoked by stimulation of rostral regions of the posterior IC (Verberne and Owens, 1998; Marins et al., 2016). Thus, restraint stress seems to activate excitatory output in the rostral posterior IC to centers in the medulla regulating the sympathetic activity to blood vessels.

The control of pressor response by rostral posterior IC seems to occur *via* indirect connections with autonomic centers in the medulla controlling the sympathetic activity. Indeed, a recent study identified neurons in the rostral posterior region projecting to the caudal ventrolateral medulla (CVLM), but not to rostral ventrolateral medulla (RVLM) (Marins et al., 2016). The site in rostral posterior region in which the projections to CVLM were identified (IA 10.5 mm) is slightly more rostral in relation to that in which we identified an involvement in pressor response to restraint stress (IA 9.7 mm) (Marins et al., 2016). However, stimulation of both regions in rostral posterior IC evoked the same pattern of cardiovascular response (i.e., pressor response) in rats under resting conditions (Yasui et al., 1991; Marins et al., 2016), thus indicating that the sites explored in the present study might also project to CVLM. GABAergic neurons in the CVLM inhibit premotor sympathetic neurons located in the RVLM. In this sense, projections to infralimbic cortex, basolateral and central amygdala, bed nucleus of the stria terminalis (BNST), and lateral hypothalamus (LH) were identified from sites in the rostral posterior IC in which stimulation evoked pressor response in animals at rest (Yasui et al., 1991; Verberne and Owens, 1998). More recent evidence indicated that these projections are mainly glutamatergic (Centanni et al., 2019; McGinnis et al., 2020; Girven et al., 2021), and some of these structures (e.g., LH) have already been implicated in cardiovascular regulation by IC under resting conditions (Cechetto and Chen, 1990; Oppenheimer et al., 1992). Furthermore, all these limbic structures are involved in the regulation of stress-evoked cardiovascular changes (Tavares et al., 2009; Deolindo et al., 2013; Oscar et al., 2015; Barretto-de-Souza et al., 2018; Gomes-de-Souza et al., 2019; Silva et al., 2020). Therefore, these regions might constitute intermediate sites connecting the rostral posterior IC to autonomic centers in the medulla for control of the pressor response during aversive threats.

The decreased number of Fos-positive cells in the caudal posterior region of the IC following restraint stress exposure indicates that the control of restraint-evoked tachycardia by this region is mediated by a reduction in local neuronal activity. Previous studies provided evidence that stimulation of caudal regions of the posterior IC evoked bradycardia in rats at rest *via* inhibition of cardiac sympathetic activity (Oppenheimer and Cechetto, 1990, Oppenheimer and Cechetto, 2016). Therefore, the role of the caudal posterior IC in restraint-evoked tachycardia seems to be mediated by a decrease in the activity of neuronal output inhibiting cardiac sympathetic activity. This idea is supported by the evidence obtained in animals subjected to lesion of the posterior IC, including the caudal regions, of a tonic inhibitory influence on sympathetic activity regulating the cardiovascular function (Butcher and Cechetto, 1995a,b; Marins et al., 2020). Moreover, removal of this sympathetic inhibitory influence obtained by neuronal lesion of the posterior IC (including caudal regions) enhanced the HR response to air-jet stress (Cheung et al., 1997).

Regarding the neural network involved in the control of restraint tachycardia by caudal posterior IC, previous evidence indicated a similar projection pattern of the subregions along the posterior IC (Yasui et al., 1991; Verberne and Owens, 1998). Therefore, although Marins et al. (2016) have not explored the connections of caudal posterior IC, regulation of tachycardia during restraint might be mediated by a decrease in the activity of neurons in the caudal posterior region projecting to CVLM. Besides, such as discussed for the control by rostral posterior region, regulation by caudal posterior IC might also be mediated by connection with limbic intermediate sites, which in turn projects to autonomic centers in the medulla controlling the cardiac sympathetic activity. Considering the evidence that projections from the IC are mainly glutamatergic (Centanni et al., 2019; McGinnis et al., 2020; Girven et al., 2021), the involvement in HR response to restraint *via* a decrease in local neuronal activation indicates that this control is mediated by connection with limbic structures playing an inhibitory role in restraint-evoked tachycardia. Thus, the decrease in the activity of IC glutamatergic neurons projecting to limbic regions inhibiting the HR response to stress would decrease the neuronal activity in these regions, which in turn would contribute to the HR increase during restraint stress. In this sense, the LH and BNST might constitute prominent limbic intermediate centers since previous studies demonstrated that activation of neurons in these regions inhibits restraint-evoked HR increase (Crestani et al., 2009; Deolindo et al., 2013; Gomes-de-Souza et al., 2019).

In summary, the findings documented in this study suggest a site-specific regulation of cardiovascular changes to restraint stress along the rostrocaudal axis of the posterior IC. Our data indicate that neuronal activation in the rostral region of the posterior IC seems to be involved in the arterial pressure increase during aversive threats. Besides, the present findings provide evidence that a decrease in neuronal activity in the caudal region of the posterior IC contributes to tachycardiac response during restraint stress. Finally, decreased neuronal activity in the anterior IC seems to be important in the control of responses other than cardiovascular adjustments during aversive threats.

## Data Availability Statement

The original contributions presented in the study are included in the article/supplementary material, further inquiries can be directed to the corresponding author/s.

## Ethics Statement

The animal study was reviewed and approved by Ethical Committee for Use of Animals of the School of Pharmaceutical Sciences—UNESP.

## Author Contributions

RT and LG-d-S: conceptualization, methodology, formal analysis, investigation, writing—original draft, and visualization. LR-S: formal analysis, investigation, writing—review and editing. CC: conceptualization, methodology, resources, data curation, writing—review and editing, visualization, supervision, project administration, and funding acquisition. All authors contributed to the article and approved the submitted version.

## Conflict of Interest

The authors declare that the research was conducted in the absence of any commercial or financial relationships that could be construed as a potential conflict of interest.

## Publisher’s Note

All claims expressed in this article are solely those of the authors and do not necessarily represent those of their affiliated organizations, or those of the publisher, the editors and the reviewers. Any product that may be evaluated in this article, or claim that may be made by its manufacturer, is not guaranteed or endorsed by the publisher.
